# Preset-Time Convergence Fuzzy Zeroing Neural Network for Chaotic System Synchronization: FPGA Validation and Secure Communication Applications

**DOI:** 10.3390/s25175394

**Published:** 2025-09-01

**Authors:** Liang Xiao, Lv Zhao, Jie Jin

**Affiliations:** 1 Sanya Institute of Hunan University of Science and Technology, Sanya 572024, China; 22010401004@mail.hnust.edu.cn; 2 School of Information and Electrical Engineering, Hunan University of Science and Technology, Xiangtan 411201, China; 3 School of Information Engineering, Changsha Medical University, Changsha 410219, China

**Keywords:** chaotic system synchronization, aperiodic excitations, zeroing neural network (ZNN), Takagi–Sugeno fuzzy control, secure communication, field-programmable gate array (FPGA)

## Abstract

Chaotic systems, characterized by extreme sensitivity to initial conditions and complex dynamical behaviors, exhibit significant potential for applications in various fields. Effective control of chaotic system synchronization is particularly crucial in sensor-related applications. This paper proposes a preset-time fuzzy zeroing neural network (PTCFZNN) model based on Takagi–Sugeno fuzzy control to achieve chaotic synchronization in aperiodic parameter exciting chaotic systems. The designed PTCFZNN model accurately handles the complex dynamic variations inherent in chaotic systems, overcoming the challenges posed by aperiodic parameter excitation to achieve synchronization. Additionally, field-programmable gate array (FPGA) verification experiments successfully implemented the PTCFZNN-based chaotic system synchronization control on hardware platforms, confirming its feasibility for practical engineering applications. Furthermore, experimental studies on chaos-masking communication applications of the PTCFZNN-based chaotic system synchronization further validate its effectiveness in enhancing communication confidentiality and anti-jamming capability, highlighting its important application value for securing sensor data transmission.

## 1. Introduction

Since Edward Lorenz discovered the chaos phenomenon more than sixty years ago [1], it has triggered a wide range of research in the academic community. Chaos synchronization, as a core topic in the field of chaos control, demonstrates significant application value in areas such as secure communication [2,3,4], electronic systems [5,6,7], and ecological systems [8]. Particularly in the domain of sensor networks [9], chaotic systems, with their unparalleled sensitivity to initial states and intricate nonlinear dynamics, offer novel potential solutions for the encryption and protection of sensitive data transmission. For example, utilizing an electro-optical hybrid entropy source, Shao and Fu [10] successfully implemented and validated a chaos synchronization scheme, demonstrating its feasibility over significant distances. This scheme achieved high-level synchronization over a distance of 200 km and has the potential to further extend the distance. In this scheme, the chaos synchronization induced by digital signals shows good robustness to long-distance transmission distortion; Rahman and Jasim [11] developed a novel fractional-order chaotic system capable of achieving master–slave synchronization despite parameter uncertainties, demonstrating significant promise for high-security communication applications resistant to eavesdropping attacks. Beyond providing physical-layer encryption for sensor data, chaotic synchronization also enhances performance in signal recovery, fault detection, system identification, and redundant fault tolerance, positioning itself as a key enabling technology for next-generation intelligent sensor networks.

In order to synchronize chaotic systems, the academic community has developed a variety of innovative control strategies, including adaptive backstepping techniques [12], neurodynamic approaches [13], active control frameworks [14], and sliding mode strategies [15]. Among them, neurodynamic methods have increasingly become an important research tool for complex system control due to their outstanding parallel processing capabilities, distributed information storage characteristics, and excellent adaptive learning mechanisms. As a typical recurrent neural network, the ZNN framework proposed by Zhang’s team [16] has been widely used in the field of numerical computing, successfully solving typical mathematical problems such as matrix inverse operation [17,18,19,20], linear algebraic equations [21,22,23,24], Sylvester equations [25,26,27], quadratic programming [28,29,30], and image encryption [31,32], fully verifying the multi-functional characteristics of the model. In the field of chaotic system synchronization, Zhao et al. [33] developed an adaptive parameter ZNN for FPGA-implemented chaotic synchronization, while Fang et al. [34] designed a complex-valued time-varying zeroing neural network model that successfully realized synchronization in complex chaotic systems.

At the same time, since Zadeh’s introduction of fuzzy set theory in 1965 [35], fuzzy logic control has been developed and widely used in various fields [36,37,38]. However, in terms of chaotic system synchronization, there is still little research on fuzzy neural networks for chaotic system synchronization, and the development of a fuzzy ZNN model for chaotic system synchronization still needs to be explored.

Despite these advances, several critical limitations remain in the current research on chaos synchronization: (i) Most existing methods focus on chaotic systems with constant or periodically varying parameters, leaving a significant gap in handling systems with aperiodic parameter excitations, which are common in real-world environments due to unpredictable disturbances and environmental fluctuations. (ii) While ZNN-based approaches offer fast convergence, their performance often degrades under noisy conditions, and few studies have systematically addressed noise suppression in chaotic synchronization. (iii) The convergence time of many neural network-based controllers is highly dependent on initial conditions, making it difficult to guarantee performance in time-critical applications such as secure communication. (iv) Although simulation results are promising, hardware-level validation—especially on reconfigurable platforms like FPGAs—is still limited, hindering the transition from theoretical models to practical implementations.

To address these challenges, this paper proposes a novel preset-time convergence fuzzy zeroing neural network (PTCFZNN) model by integrating Takagi–Sugeno (T-S) fuzzy logic with the ZNN framework. The integration of fuzzy logic enables dynamic adjustment of the convergence rate based on synchronization error, significantly enhancing adaptability and robustness. The proposed PTCFZNN achieves fixed-time convergence independent of initial states, ensures strong noise tolerance, and effectively handles chaotic systems under aperiodic parameter excitations. Furthermore, we implemented the PTCFZNN on an FPGA platform, validating its real-time feasibility and hardware efficiency. We also demonstrate its application in chaos-masking secure communication, showing superior signal recovery and confidentiality. Additionally, the main contributions of this paper include

- Proposing a PTCFZNN model based on T-S fuzzy control, which significantly improves convergence speed and anti-interference capabilities through the design of a fuzzy time-varying convergence factor and a novel activation function.
- Providing theoretical proof of the PTCFZNN’s global stability and deriving an explicit upper bound for convergence time.
- Validating the superior performance of the PTCFZNN model through simulation experiments and FPGA hardware implementation, both in noise-free and various noisy environments.
- Innovatively applying chaos-masking technology to secure communication experiments, demonstrating PTCFZNN’s practical potential in photoelectric signal encryption and transmission.

The structure of this paper is organized as follows: Section 2 introduces the synchronization problem of chaotic systems with aperiodic parameter excitation; Section 3 details four ZNN models, including the proposed PTCFZNN; Section 4 provides theoretical analysis of PTCFZNN’s stability, convergence, and robustness; Section 5 validates the model’s performance through simulations and chaos-masking experiments; Section 6 presents FPGA hardware implementation results; and Section 7 summarizes the work and outlines future research directions.

## 2. Problem Description and Preliminaries

### 2.1. Synchronization Problem of Chaotic Systems with Aperiodic Parameter Excitation

The master–slave synchronization challenge in aperiodically parametrically excited chaotic systems is defined by the master chaotic system dynamics, and the master chaotic system is defined below:

(1) $${\dot{x}}_{m}\left(t\right)=f_{m}\left(x_{m}\left(t\right),\sigma \left(t\right)\right)+\Delta n\left(t\right)$$

where $x_{m}\left(t\right)={\left[x_{m1}\left(t\right),x_{m2}\left(t\right),\ldots ,x_{mn}\left(t\right)\right]}^{T}\in R^{n}$ is an *n*-dimensional state vector. The nonlinear function $f_{m}\left(\cdot \right):R^{n}\to R^{n}$ defines the inherent dynamic characteristics of the system. In addition, σ(t) represents the non-periodic time-varying parameter. While $\Delta n\left(t\right)\in R^{n}$ represents the external noise interference.

The slave system is described by the following equation:

(2) $${\dot{x}}_{s}\left(t\right)=f_{s}\left(x_{s}\left(t\right),\sigma \left(t\right)\right)+u\left(t\right)$$

where $x_{s}\left(t\right)={\left[x_{s1}\left(t\right),x_{s2}\left(t\right),\ldots ,x_{sn}\left(t\right)\right]}^{T}\in R^{n}$ is an *n*-dimensional state vector. The nonlinear function $f_{s}\left(\cdot \right):R^{n}\to R^{n}$ defines the inherent dynamic characteristics of the system. In addition, σ(t) represents the non-periodic time-varying parameter. Furthermore, u(t) denotes the control vector designed to drive the slave system to synchronize with the master system.

Based on the above analysis of the synchronization process, the synchronization error can be defined in the form below:

(3) $$e\left(t\right)=x_{m}\left(t\right)-x_{s}\left(t\right)$$

Our goal is to design an appropriate controller ensuring the following condition holds:

(4) $$\lim_{t\to \infty }∥e\left(t\right)∥=0$$

### 2.2. Mathematical Knowledge

**Definition 1**  ([39])**.** *For any positive real pair $\lambda_{1},\lambda_{2}$, the Beta function is introduced by the integral representation below:*

(5) $$B\left(\lambda_{1},\lambda_{2}\right)=\int_{0}^{1}x^{\lambda_{1}-1}{\left(1-x\right)}^{\lambda_{2}-1}dx$$

**Definition 2**  ([39])**.** *For any real β satisfying 0<β≤1, the incomplete Beta function is introduced by the integral representation below:*

(6) $$I\left(\beta ,\lambda_{1},\lambda_{2}\right)=\frac{1}{B\left(\lambda_{1},\lambda_{2}\right)}\int_{0}^{\beta }x^{\lambda_{1}-1}{\left(1-x\right)}^{\lambda_{2}-1}dx$$

## 3. Zeroing Neural Network Models

To address the challenge of achieving synchronized dynamics in chaotic systems under aperiodic parametric excitation, four models, including the conventional ZNN (CZNN) model, super-exponential ZNN (SEZNN) model, disturbance suppression ZNN (DSZNN) model, and the proposed preset-time fuzzy ZNN (PTCFZNN) model, are constructed and introduced in this section.

### 3.1. CZNN Model

In Ref. [40], Li et al. proposed the CZNN model, realizing chaos synchronization by constructing an error dynamic evolution equation. Its core idea is to transform the convergence process of the synchronization error as the solution of a differential equation. The CZNN dynamics are represented by the following expression:

(7) $$\dot{e}\left(t\right)=-Y\Gamma \left(e\left(t\right)\right)$$

where Y≥0 is the fixed convergence factor that determines the adjustment rate of the system, and $\Gamma \left(\cdot \right)$ is the linear activation function, that is, $\begin{array}{c} \Gamma \left(x\right)=x \end{array}$. Substituting the master–slave system dynamic Equations (1) and (2) into it, the CZNN model for the synchronization of chaotic systems with aperiodic parameter excitations can be obtained:

(8) $$\begin{array}{c} f_{m}\left(x_{m}\left(t\right),\sigma \left(t\right)\right)+\Delta n\left(t\right)-f_{s}\left(x_{s}\left(t\right),\sigma \left(t\right)\right)-u\left(t\right)\\ =-Y\Gamma \left(x_{r}\left(t\right)-x_{s}\left(t\right)\right) \end{array}$$

### 3.2. SEZNN Model

In Ref. [41], Chen et al. proposed the SEZNN model. The SEZNN model improves convergence speed through

(9) $$\dot{e}\left(t\right)=-Y\exp\left(t\right)\Gamma \left(e\left(t\right)\right)$$

where the fixed convergence factor Y≥0 and the time-varying gain term exp(t) work together to improve the convergence speed of the system, and the function $\Gamma \left(\cdot \right)$ denotes a linear activation function, defined as $\begin{array}{c} \Gamma \left(x\right)=x \end{array}$. Consequently, the SEZNN approach is formulated as follows:

(10) $$\begin{array}{c} f_{m}\left(x_{m}\left(t\right),\sigma \left(t\right)\right)+\Delta n\left(t\right)-f_{s}\left(x_{s}\left(t\right),\sigma \left(t\right)\right)-u\left(t\right)\\ =-Y\exp\left(t\right)\Gamma \left(x_{r}\left(t\right)-x_{s}\left(t\right)\right) \end{array}$$

### 3.3. DSZNN Model

In order to enhance the noise suppression ability, in Ref. [42], Chen et al. proposed the disturbance suppression ZNN (DSZNN) model, and its dynamic equation was improved as

(11) $$\dot{e}\left(t\right)=-Y\exp\left(\kappa t\right)\Gamma \left(e\left(t\right)\right)+\varpi \left(t\right)$$

In the formula, Y>0 and κ>0. The nonlinear activation mapping is accordingly constructed in the form

(12) $$\Gamma \left(x\right)={\left(\alpha |x\right|}^{p}+{\beta |x|}^{1/p}+{\eta |x|}^{\tau })\cdot \mathrm{sign}\left(x\right)$$

with parameters α,β,η>0 and exponents p,τ>0. Subsequently, the DSZNN model is derived to address the synchronization of chaotic systems with aperiodic parameter excitations, as detailed below:

(13) $$\begin{array}{c} f_{m}\left(x_{m}\left(t\right),\sigma \left(t\right)\right)+\Delta n\left(t\right)-f_{s}\left(x_{s}\left(t\right),\sigma \left(t\right)\right)-u\left(t\right)\\ =-Y\exp\left(\kappa t\right)\Gamma \left(x_{r}\left(t\right)-x_{s}\left(t\right)\right) \end{array}$$

### 3.4. The Proposed PTCFZNN Model

In this subsection, a method for detailing the performance of the proposed PTCFZNN model is introduced.

This study addresses the challenging problem of aperiodic parameter chaos synchronization by innovatively combining the Takagi–Sugeno fuzzy logic system (TSFLS) with the ZNN method. Through the design of an error-based dynamic adjustment mechanism, the optimization and improvement of the system’s convergence behavior are achieved.

The steps for the TSFLS to generate the fuzzy parameter *v* are presented below.

1. Fuzzification: Fuzzification is the primary step in constructing an effective control system. The bell-shaped membership function (Gbellmf) is used to achieve the mapping from precise quantities to fuzzy sets. Its expression is
  (14) $$\mu \left(\xi \right)=\frac{1}{1+{\left|\frac{\xi -c}{a}\right|}^{2b}}$$
  where ξ is the input, and a,b,c are constants. To increase the system’s sensitivity to errors, the fuzzy system takes the absolute synchronization error ξ(t)=|e(t)|∈[0,∞) as its input, allowing for a more precise characterization of the deviation from the desired trajectory.
2. Fuzzy Inference Engine: The rule base serves as a central component of the fuzzy logic system, and its design significantly influences the overall control performance. Differentiated linear output functions are adopted when the system error is in different intervals.
  (15) $$\begin{array}{cc} \; & R^{1}:\;\mathrm{IF}\;e=ZO,\;\mathrm{THEN}\;v_{1}=6.\\ \; & R^{2}:\;\mathrm{IF}\;e=PS,\;\mathrm{THEN}\;v_{2}=2e+4.\\ \; & R^{3}:\;\mathrm{IF}\;e=PM,\;\mathrm{THEN}\;v_{3}=3e+3.5.\\ \; & R^{4}:\;\mathrm{IF}\;e=PB,\;\mathrm{THEN}\;v_{4}=4.5e+3. \end{array}$$
  where *ZO*, *PS*, *PM*, and *PB* denote fuzzy sets corresponding to zero, small, medium, and large error magnitudes, respectively.
3. Defuzzification: Defuzzification is a crucial step in converting the results of fuzzy inference into actual control parameters. Considering the sensitivity of the chaotic system to parameter changes, the weighted average method (wtaver) is used as the defuzzification operator, which can normalize the rule weights and suppress drastic changes. The calculation formula is as follows:
  (16) $$v=\frac{\sum_{j=1}^{4}\omega_{j}v_{j}}{\sum_{j=1}^{4}\omega_{j}}$$

Based on this, the fuzzy parameter *v* is derived, and a novel fuzzy time-varying convergence factor is constructed as follows:

(17) $$Y\left(t\right)={\left(\rho_{1}\mathrm{arccot}\left(t\right)+\rho_{2}t\right)}^{v}+\sigma$$

where $\rho_{1},\rho_{2},\sigma >0$.

At the same time, the following novel activation function is designed:

(18) $$\Gamma \left(x\right)=\exp\left(-|x|\right)\left(\alpha_{1}\exp\left(\epsilon |x|\right)+\alpha_{2}\exp\left(\varepsilon |x|\right)\right)sign\left(x\right)+\alpha_{3}x$$

where $\alpha_{1}>0,\alpha_{2}>0,\alpha_{3}>0,1>\epsilon >0,$ and ε≥1.

Accordingly, the following PTCFZNN model is established to achieve synchronization in chaotic systems with aperiodic parameter excitations:

(19) $$f_{m}\left(x_{m}\left(t\right),\sigma \left(t\right)\right)+\Delta n\left(t\right)-f_{s}\left(x_{s}\left(t\right),\sigma \left(t\right)\right)-u\left(t\right)=-Y\left(t\right)\Gamma \left(x_{r}\left(t\right)-x_{s}\left(t\right)\right)$$

As a result, the formulation of the PTCFZNN-based controller follows directly from Equation (19):

(20) $$u\left(t\right)=f_{m}\left(x_{m}\left(t\right),\sigma \left(t\right)\right)+f_{s}\left(x_{s}\left(t\right),\sigma \left(t\right)\right)+Y\left(t\right)\Gamma \left(x_{r}\left(t\right)-x_{s}\left(t\right)\right)$$

## 4. Theoretical Analysis of the PTCFZNN Model

### 4.1. Stability Analysis

**Proof.**  Firstly, the evolution formula for the *i*-th elements is presented as follows:

(21) $${\dot{e}}_{i}\left(t\right)=-Y\left(t\right)\Gamma \left(e_{i}\left(t\right)\right)$$

where i=1,2,…,n, and $e_{i}\left(t\right)$ is an element of e(t).

A Lyapunov function is constructed as follows:

(22) $$\Xi_{i}\left(t\right)=e_{i}^{2}\left(t\right)/2$$

Given that $\Xi_{i}$ is a monotonic odd function, we can infer that

(23) $${\dot{\Xi }}_{i}\left(t\right)\left\{\begin{array}{cc} <0, & e_{i}\left(t\right)>0\\ =0, & e_{i}\left(t\right)=0\\ <0, & e_{i}\left(t\right)<0 \end{array}\right.$$

The Lyapunov stability analysis theory in Ref. [43] states that if a continuously differentiable function $\Xi_{i}\left(t\right)$ (Lyapunov function) is positive definite and its time derivative ${\dot{\Xi }}_{i}\left(e\left(t\right)\right)$ is negative semi-definite along the system trajectories, then the equilibrium point e(t)=0 is stable in the sense of Lyapunov. Here, $\Xi_{i}\left(t\right)=e_{i}^{2}\left(t\right)/2$ satisfies these conditions, confirming global asymptotic stability of the error system under the PTCFZNN control. Thus, the stability proof is completed. □

### 4.2. Convergence Analysis

**Theorem 1.**  *In a noise-free environment and under any initial conditions, if the slave chaotic system is controlled by PTCFZNN, through variable substitution and the derivation of integral inequalities, an explicit upper limit for the convergence interval is derived as*

(24) $$t_{p}\leq \frac{1}{\sigma }\frac{1}{\alpha_{1}\left(\varepsilon -\epsilon \right)}{\left(\frac{\alpha_{1}}{\alpha_{2}}\right)}^{\varpi_{\epsilon }}B\left(\varpi_{\varepsilon },\varpi_{\epsilon }\right)I\left(\frac{\alpha_{1}}{\alpha_{2}+\alpha_{1}},\varpi_{\varepsilon },\varpi_{\epsilon }\right)$$

**Proof of Theorem 1.**  If it can be verified that the subsystem can converge within the predefined time range, then Theorem 1 will be verified. To establish the predefined-time convergence property of PTCFZNN, the following Lyapunov function is designed:

(25) $$V_{i}\left(t\right)=\left|e_{i}\left(t\right)\right|$$

Because $|e_{i}\left(t\right)|=e_{i}\left(t\right)sign\left(e_{i}\left(t\right)\right)$ and through the exploitation of the odd symmetry of Γ(·), the derivative of $V_{i}\left(t\right)$ becomes

(26) $$\begin{array}{cc} \frac{d|e_{i}\left(t\right)|}{\;dt} & =\frac{\;d|e_{i}\left(t\right)|}{\;de_{i}\left(t\right)}\frac{\;de_{i}\left(t\right)}{\;dt}\\ & =-\mathrm{sign}\left(e_{i}\left(t\right)\right)Y\left(t\right)\Gamma \left(e_{i}\left(t\right)\right)\\ & =-Y\left(t\right)\Gamma \left(\left|e_{i}\left(t\right)\right|\right) \end{array}$$

Substituting (18) into (26), we can obtain

(27) $$\begin{array}{cc} {\dot{V}}_{i}\left(t\right) & =-Y\left(t\right)((\exp\left(-V_{i}\right)\left(\alpha_{1}\exp\left(\epsilon V_{i}\right)+\alpha_{2}\exp\left(\varepsilon V_{i}\right))+\alpha_{3}V_{i}\right)\\ \; & \leq -Y\left(t\right)\exp\left(-V_{i}\right)\left(\alpha_{1}\exp\left(\epsilon V_{i}\right)+\alpha_{2}\exp\left(\varepsilon V_{i}\right)\right) \end{array}$$

By integrating both sides of the above formula and substituting the variable $\gamma =\exp\left(V_{i}\right)$, Equation (27) can be transformed into

(28) $$\int_{0}^{t_{p}}Y\left(t\right)dt\leq \int_{1}^{\infty }\frac{1}{\alpha_{1}\gamma^{\epsilon }+\alpha_{2}\gamma^{\varepsilon }}d\gamma$$

The left side of Formula (28) has

(29) $$\begin{array}{cc} \int_{0}^{t_{p}}Y\left(t\right)dt & =\int_{0}^{t_{p}}\left({\left(\rho_{1}\mathrm{arccot}\left(t\right)+\rho_{2}t\right)}^{v}+\sigma \right)dt\\ & >\int_{0}^{t_{p}}\left({\left(\rho_{2}t\right)}^{v}+\sigma \right)dt>\int_{0}^{t_{p}}\sigma dt \end{array}$$

The right side of Equation (28) has

(30) $$\begin{array}{cc} \int_{1}^{+\infty }\frac{1}{\alpha_{1}\gamma^{\epsilon }+\alpha_{2}\gamma^{\varepsilon }}d\gamma & =\int_{1}^{+\infty }\frac{\alpha_{1}^{-1}\gamma^{-\epsilon }}{\frac{\alpha_{2}}{\alpha_{1}}\gamma^{\epsilon -\varepsilon }+1}d\gamma \end{array}$$

Let $\tau =\alpha_{1}/\left(\alpha_{2}\gamma^{\varepsilon -\epsilon }+1\right)$. Its asymptotic behavior is as follows: when γ→1, the limit of τ is $\alpha_{1}/\left(\alpha_{2}+\alpha_{1}\right)$; when γ→0, τ approaches positive infinity. Furthermore, by substituting the variable τ, we can inversely solve to obtain

(31) $$d\gamma =\frac{1}{\varepsilon -\epsilon }{\left(\frac{\alpha_{1}}{\alpha_{2}}\right)}^{1/\left(\varepsilon -\epsilon \right)}{\left(1/\tau -1\right)}^{1/\left(\varepsilon -\epsilon \right)-1}\left(-\tau^{-2}\right)d\tau$$

Let $\varpi_{\varepsilon }=\left(\varepsilon -1\right)/\left(\varepsilon -\epsilon \right)$ and $\varpi_{\epsilon }=\left(1-\epsilon \right)/\left(\varepsilon -\epsilon \right)$. Using Definitions 1 and 2 and combining Equations (29)–(31), we have

(32) $$\begin{array}{cc} t_{p} & \leq \frac{1}{\sigma }\frac{1}{\alpha_{1}\left(\varepsilon -\epsilon \right)}{\left(\frac{\alpha_{1}}{\alpha_{2}}\right)}^{\frac{1-\epsilon }{\varepsilon -\epsilon }}\int_{0}^{\frac{\alpha_{1}}{\alpha_{2}+\alpha_{1}}}\gamma^{\frac{\varepsilon -1}{\varepsilon -\epsilon }-1}{\left(1-\gamma \right)}^{\frac{1-\epsilon }{\varepsilon -\epsilon }-1}d\gamma \\ & \leq \frac{1}{\sigma }\frac{1}{\alpha_{1}\left(\varepsilon -\epsilon \right)}{\left(\frac{\alpha_{1}}{\alpha_{2}}\right)}^{\varpi_{\epsilon }}B\left(\varpi_{\varepsilon },\varpi_{\epsilon }\right)I\left(\frac{\alpha_{1}}{\alpha_{2}+\alpha_{1}},\varpi_{\varepsilon },\varpi_{\epsilon }\right) \end{array}$$

The proof is complete. □

The result shows that the convergence time of the system mainly depends on design parameters $\alpha_{1},\alpha_{2}$ and fuzzy output *v* and is independent of the initial state. This characteristic overcomes the defect of the initial sensitivity of the traditional models.

### 4.3. Robustness Analysis

**Theorem 2.**  *Assume there exists an unknown bounded noise $|\Delta n\left(t\right)|\leq \sigma \alpha_{3}\left|e_{i,j}\left(t\right)\right|$. Under the action of the PTCFZNN controller, it can be ensured that the slave chaotic system will synchronize with the master chaotic system within the preset time interval.*

**Proof of Theorem 2.**  First, the design formula of the PTCFZNN disturbed by noise can be expressed as follows:

(33) $${\dot{e}}_{i,j}\left(t\right)=-Y\left(t\right)\Gamma \left(e_{i,j}\left(t\right)\right)+\Delta n\left(t\right)$$

To analyze the convergence of the system, the maximum synchronization error component is defined as $m_{\left(t\right)}=\max e_{i}$. When $m_{\left(t\right)}=0$, all error components $e_{i}\left(t\right)$ converge to 0, resulting in complete system synchronization. When $m_{\left(t\right)}\neq 0$, its derivative satisfies

(34) $$\begin{array}{cc} \frac{d|m(t)|}{dt} & =\mathrm{sign}\left(m\left(t\right)\right)\cdot \dot{m}\left(t\right)\\ & =-Y\left(t\right)\exp\left(-|m\left(t\right)|\right)\left(\alpha_{1}\exp\left(\epsilon |m\left(t\right)|\right)+\alpha_{2}\exp\left(\epsilon |m\left(t\right)|\right)\right)\\ & \;+\mathrm{sign}\left(m\left(t\right)\right)\Delta n\left(t\right)-Y\left(t\right)\alpha_{3}\left|m\left(t\right)\right| \end{array}$$

Under the noise constraint, there is

(35) $$Y\left(t\right)\alpha_{3}|m\left(t\right)|-|\Delta n\left(t\right)|\geq \sigma \alpha_{3}|m\left(t\right)|-\sigma \alpha_{3}\left|m\left(t\right)\right|=0$$

Through the combination of Equations (34) and (35), the error dynamic equation can be simplified as follows:

(36) $$\frac{d|m(t)|}{dt}\leq -\frac{1}{Y(t)}\left(\exp\left(-|m\left(t\right)|\right)\left(\alpha_{1}\exp\left(\epsilon |m\left(t\right)|\right)+\alpha_{2}\exp\left(\varepsilon |m\left(t\right)|\right)\right)\right)$$

At this time, the error dynamic equation is consistent with the noiseless case in Theorem 1, so the upper bound of the convergence time remains unchanged. □

The preceding theoretical analysis reveals the superiority of the PTCFZNN model through three representative aspects: stability, convergence rate, and robustness, providing a theoretical guarantee for chaotic synchronization control in complex environments.

## 5. Simulation Verification

### 5.1. The Chen Hyper-Chaotic System

In this subsection, the Chen hyper-chaotic system is adopted to verify the performance of the proposed PTCFZNN model for chaotic system synchronization.

The expression of the original Chen hyper-chaotic system with aperiodic parameter excitations is as follows:

(37) $$\left\{\begin{array}{c} {\dot{x}}_{1}\left(t\right)=\left(a+\varkappa_{1}z_{1}\right)\left(x_{2}\left(t\right)-x_{1}\left(t\right)\right)+x_{4}\left(t\right)\\ {\dot{x}}_{2}\left(t\right)=\left(d+\varkappa_{2}z_{2}\right)x_{1}\left(t\right)-x_{1}\left(t\right)x_{3}\left(t\right)+cx_{2}\left(t\right)\\ {\dot{x}}_{3}\left(t\right)=x_{1}\left(t\right)x_{2}\left(t\right)-\left(b+\varkappa_{3}z_{3}\right)x_{3}\left(t\right)\\ {\dot{x}}_{4}\left(t\right)=x_{2}\left(t\right)x_{3}\left(t\right)+\left(r+\varkappa_{4}z_{4}\right)x_{4}\left(t\right) \end{array}\right.$$

where a=35, b=3, c=12, d=7, r=0.16, and $\varkappa_{1}=\varkappa_{2}=\varkappa_{3}=\varkappa_{4}=0.001$ is the perturbation intensity. Moreover, $z_{i}$ (i=1,2,3,4) denotes the parameter perturbations and is provided by the following four-dimensional extended Rossler chaotic system:

(38) $$\left\{\begin{array}{cc} {\dot{z}}_{1} & =-z_{2}-z_{3}+d_{1}z_{1}\\ {\dot{z}}_{2} & =z_{1}+a_{1}z_{2}+d_{1}z_{2}\\ {\dot{z}}_{3} & =b_{1}+z_{1}z_{3}-c_{1}z_{3}+d_{1}z_{3}\\ {\dot{z}}_{4} & =-z_{1}-z_{2}-z_{3}+d_{1}z4 \end{array}\right.$$

where $a_{1}=0.25$, $b_{1}=0.4$, $c_{1}=4.5$, and $d_{1}=0.001$.

Then, the master Chen hyper-chaotic system with aperiodic parameter excitations under external perturbation is as follows:

(39) $${\dot{x}}_{m}\left(t\right)=\left[\begin{array}{c} \left(a+\varkappa_{1}z_{1}\right)\left(x_{m2}\left(t\right)-x_{m1}\left(t\right)\right)+x_{m4}\left(t\right)\\ \left(d+\varkappa_{2}z_{2}\right)x_{m1}\left(t\right)-x_{m1}\left(t\right)x_{m3}\left(t\right)+cx_{m2}\left(t\right)\\ x_{m1}\left(t\right)x_{m2}\left(t\right)-\left(b+\varkappa_{3}z_{3}\right)x_{m3}\left(t\right)\\ x_{m2}\left(t\right)x_{m3}\left(t\right)+\left(r+\varkappa_{4}z_{4}\right)x_{m4}\left(t\right) \end{array}\right]+\Delta n\left(t\right)$$

The slave Chen hyper-chaotic system with the PTCFZNN-based controller (20) for synchronizing the above master Chen hyper-chaotic system (39) is described below:

(40) $${\dot{x}}_{s}\left(t\right)=\left[\begin{array}{c} \left(a+\varkappa_{1}z_{1}\right)\left(x_{s2}\left(t\right)-x_{s1}\left(t\right)\right)+x_{s4}\left(t\right)\\ \left(d+\varkappa_{2}z_{2}\right)x_{s1}\left(t\right)-x_{s1}\left(t\right)x_{s3}\left(t\right)+cx_{s2}\left(t\right)\\ x_{s1}\left(t\right)x_{s2}\left(t\right)-\left(b+\varkappa_{3}z_{3}\right)x_{s3}\left(t\right)\\ x_{s2}\left(t\right)x_{s3}\left(t\right)+\left(r+\varkappa_{4}z_{4}\right)x_{s4}\left(t\right) \end{array}\right]+u\left(t\right)$$

### 5.2. Synchronization Simulations of Chen Hyper-Chaotic Systems in Noise-Free Environment

Under ideal conditions with no noise interference, comparative experiments were conducted between the proposed PTCFZNN model and traditional CZNN, SEZNN, and DSZNN models to validate the performance advantages of PTCFZNN in synchronizing the Chen hyper-chaotic system with aperiodic parameter excitations. Specifically, the effectiveness of PTCFZNN is systematically evaluated by comparing the convergence speed, trajectory tracking accuracy, and synchronization stability across the models.

**Figure 1:**
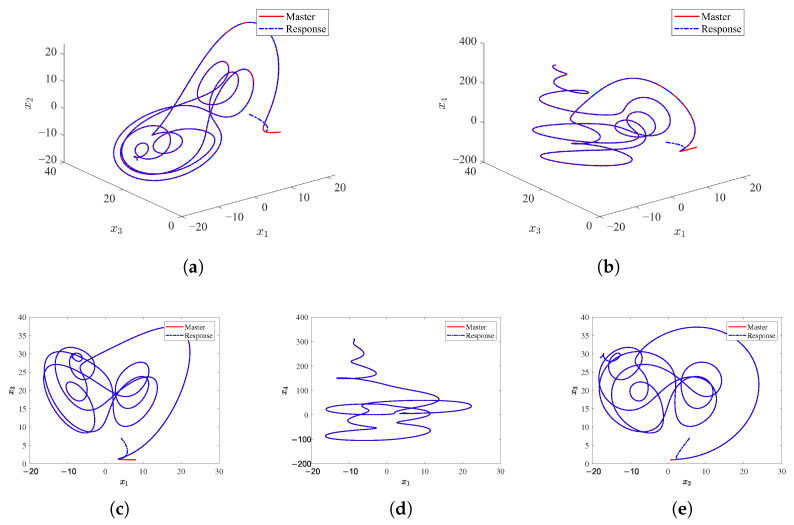
Three-dimensional and two-dimensional synchronization phase portraits of the Chen hyper-chaotic systems by the PTCFZNN controller under noise-free conditions. (**a**) $x_{1}\left(t\right)$-$x_{3}\left(t\right)$-$x_{2}\left(t\right)$. (**b**) $x_{1}\left(t\right)$-$x_{3}\left(t\right)$-$x_{4}\left(t\right)$. (**c**) $x_{1}\left(t\right)$-$x_{3}\left(t\right)$. (**d**) $x_{1}\left(t\right)$-$x_{4}\left(t\right)$. (**e**) $x_{2}\left(t\right)$-$x_{3}\left(t\right)$.

Figure 1 presents the three-dimensional (3D) and two-dimensional (2D) phase portrait projections of the master and slave systems under PTCFZNN control. The slave system trajectory (blue dashed curves) and the master system trajectory (red curves) rapidly coincide in both 3D space and 2D planes, indicating the precise tracking of the master system’s dynamic evolution by the slave system.

**Figure 2:**
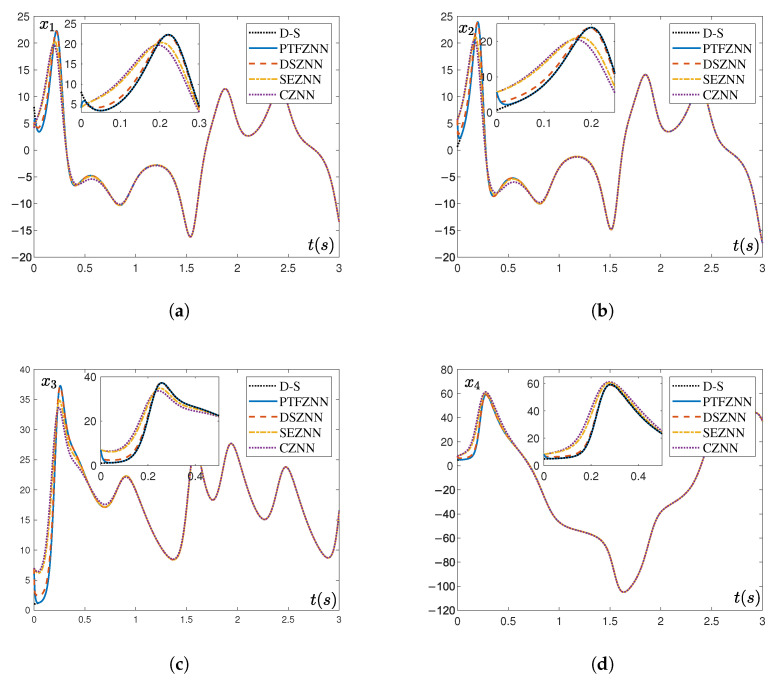
Comparison of element synchronization results of the mater and slave Chen hyper-chaotic systems using four models without noise interference. (**a**) Synchronization trajectories of $x_{m1}\left(t\right)$ and $x_{s1}\left(t\right)$. (**b**) Synchronization trajectories of $x_{m2}\left(t\right)$ and $x_{s2}\left(t\right)$. (**c**) Synchronization trajectories of $x_{m3}\left(t\right)$ and $x_{s3}\left(t\right)$. (**d**) Synchronization trajectories of $x_{m4}\left(t\right)$ and $x_{s4}\left(t\right)$.

Figure 2 further illustrates the time-domain trajectories of the master system state variables ($\left(x_{m1}\left(t\right),\ldots ,x_{m4}\left(t\right)\right)$) and the corresponding slave system state variables ($\left(x_{s1}\left(t\right),\ldots ,x_{s4}\left(t\right)\right)$) under the control of each model. The results show that all four models ultimately achieved master–slave synchronization; however, the system controlled by PTCFZNN attained state variable synchronization in significantly less time. As detailed in Table 1, the PTCFZNN-controlled system achieved a convergence time of merely 0.0404471 s in the noise-free environment, dramatically outperforming the other models: DSZNN required 0.429565 s, SEZNN required 0.74097 s, and CZNN exhibited the longest convergence time of 1.6602 s. This translates to convergence speeds for PTCFZNN that are approximately 10.6 times faster than DSZNN, 18.3 times faster than SEZNN, and 41.1 times faster than CZNN, thereby verifying the efficacy of its preset-time convergence property.

In summary, the above experimental results under noise-free conditions demonstrate that the proposed PTCFZNN model significantly outperforms existing models across all three evaluated aspects: convergence speed, tracking accuracy, and synchronization stability.

### 5.3. Synchronization Simulations of Chen Hyper-Chaotic Systems with Noise

In this subsection, to further verify the excellent robustness of the proposed PTCFZNN model for chaotic system synchronization, the CZNN, SEZNN, DSZNN, and the proposed PTCFZNN are also used for Chen hyper-chaotic system synchronization in three different noise environments (including Δn(t)=0.4t, Δn(t)=exp(−(1+t)), and Δn(t)=0.2cos(2πt)+2exp(0.3t)). The choice of noise model is motivated by practical considerations in sensor-based chaotic systems. Linear noise simulates slow, varying disturbances such as thermal drift or sensor bias. Exponential noise represents impulsive or transient interference common in communication channels. Mixed noise combines both to reflect complex real-world environments. These models are widely used in the chaos control literature to assess robustness under diverse operational conditions [33,44].

**Figure 3:**
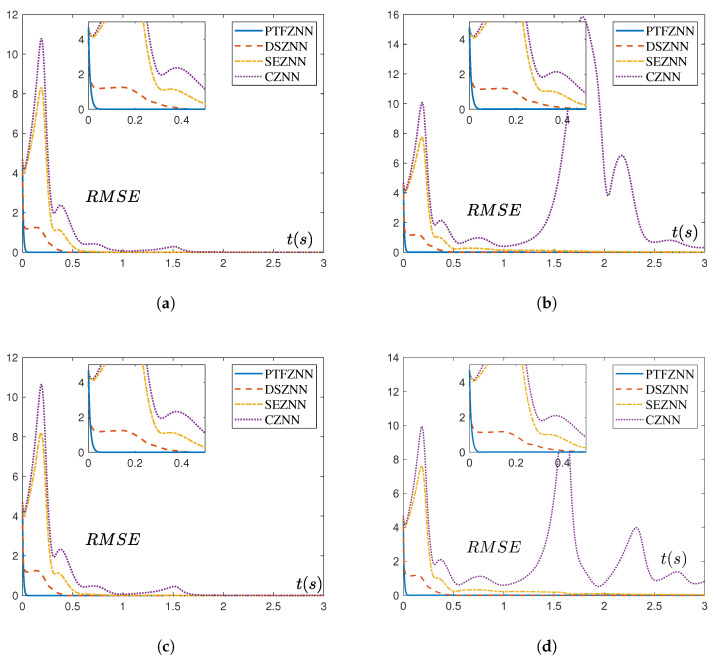
Synchronization residual errors of the four models (PTCFZNN, DSZNN, SEZNN, CZNN) for Chen hyper-chaotic system synchronization under both clean and noisy operation conditions. (**a**) Synchronization residual errors of the four models without noise. (**b**) Synchronization residual errors of the four models with linear noise. (**c**) Synchronization residual errors of the four models with exponential noise. (**d**) Synchronization residual errors of the four models with mixed noises.

As illustrated in Figure 3, the convergence behavior of the four models is presented under three distinct noise conditions. According to the data in Table 1, the PTCFZNN model demonstrates markedly superior convergence performance compared to the other three conventional models across all tested noise environments. In various noise environments, the residual of the PTCFZNN model always remains at a low level and has relatively small fluctuations. It is worth noting that the CZNN model cannot even achieve synchronous control under certain noise environments. The experiments successfully validate Theorem 3 and offer compelling evidence that the proposed PTCFZNN achieves greater robustness than the DSZNN, SEZNN, and CZNN models under various conditions.

Overall, the proposed PTCFZNN outperforms DSZNN, SEZNN, and CZNN in the synchronous control performance of aperiodic parameter chaotic systems under both clean and noisy operational conditions.

It is worth noting that while the robustness analysis in Theorem 2 is based on a bounded noise assumption commonly adopted in control theory, practical sensor and communication systems often evaluate performance using signal-to-noise ratio (SNR). Based on the simulation results in Table 1, the applied noise levels correspond approximately to an SNR range of 20–30 dB, under which the PTCFZNN model maintains fast and stable synchronization. This indicates that the proposed method is capable of operating effectively in moderately noisy environments typical of real-world sensor networks. Furthermore, performance evaluation in chaotic synchronization differs from classical digital communication systems, which typically rely on bit error rate (BER) versus $E_{b}/N_{0}$. Instead, chaotic synchronization emphasizes state tracking accuracy under noise, quantified by synchronization error. The results in Table 1 demonstrate that the proposed PTCFZNN achieves high synchronization accuracy even under significant noise, highlighting its strong robustness. This stability suggests potential applicability in chaos-based communication schemes such as CSK for reliable data recovery; however, a formal BER analysis would require a defined modulation and demodulation framework, which is reserved for future investigation.

## 6. FPGA Implementation

FPGAs, with their reconfigurability and parallel computing advantages, provide an ideal platform for the hardware implementation of complex control algorithms. To verify the applicability of the PTCFZNN model in an actual engineering environment, this study used the Xilinx xc7z020clg400-1 FPGA chip to build a hardware verification platform and observed the chaos synchronization process in real time through a digital oscilloscope. The experimental design adopted a hierarchical architecture: First, Matlab software was used to generate the state trajectory data of the master system and the slave system and convert it into a COE-format configuration file. Subsequently, the configuration data were loaded into the Block RAM of the FPGA through the JTAG interface, and a data path was constructed using eight slice registers and 32 flip-flops. Finally, the processing results were output to the oscilloscope through the IOB interface.

**Figure 4:**
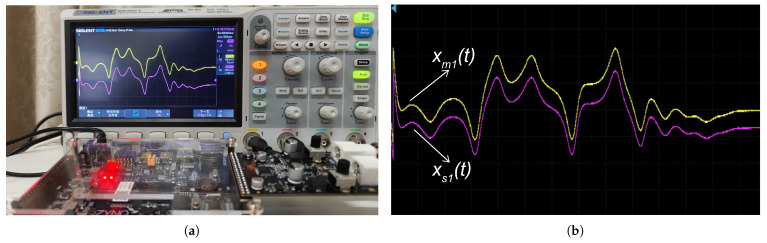

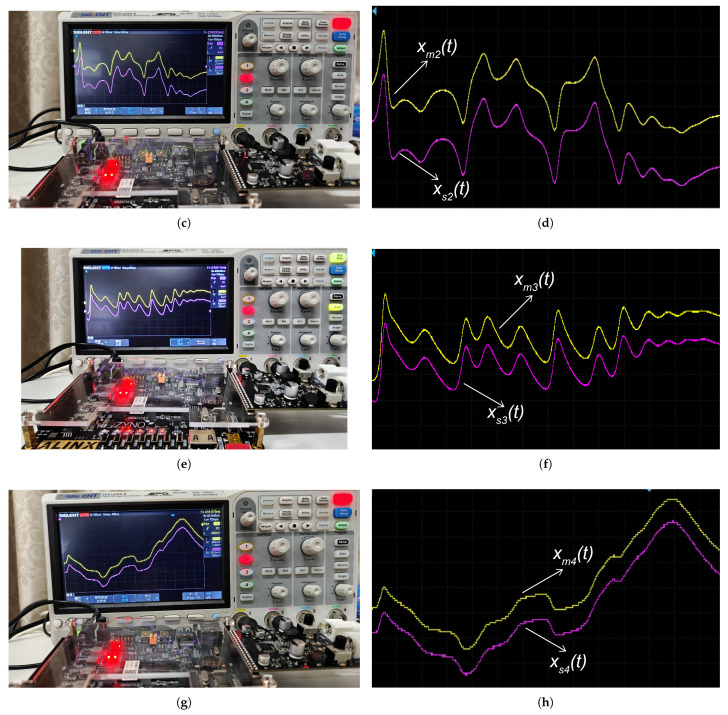
FPGA realization of PTCFZNN-driven synchronization for the Chen hyper-chaotic system with aperiodic parameter excitation. The yellow curves stand for the state trajectories of the master Chen hyper-chaotic system, and the red curves stand for the state trajectories of the slave Chen hyper-chaotic system. (**a**) Master $x_{m1}\left(t\right)$ and slave $x_{s1}\left(t\right)$ trajectories. (**b**) Synchronization dynamics between master $x_{m1}\left(t\right)$ and slave $x_{s1}\left(t\right)$. (**c**) Master $x_{m2}\left(t\right)$ and slave $x_{s2}\left(t\right)$ trajectories. (**d**) Synchronization dynamics between master $x_{m2}\left(t\right)$ and slave $x_{s2}\left(t\right)$. (**e**) Master $x_{m3}\left(t\right)$ and slave $x_{s3}\left(t\right)$ trajectories. (**f**) Synchronization dynamics between master $x_{m3}\left(t\right)$ and slave $x_{s3}\left(t\right)$. (**g**) Master $x_{m4}\left(t\right)$ and slave $x_{s4}\left(t\right)$ trajectories. (**h**) Synchronization dynamics between master $x_{m4}\left(t\right)$ and slave $x_{s4}\left(t\right)$.

Figure 4 compares the state trajectories of the master chaotic system $\left(x_{m1}\left(t\right),\ldots ,x_{m4}\left(t\right)\right)$ with corresponding slave system solutions $\left(x_{s1}\left(t\right),\ldots ,x_{s4}\left(t\right)\right)$ governed by the proposed PTCFZNN controller under noisy conditions. The yellow traces depict the master system states, while the corresponding purple traces represent the PTCFZNN-controlled slave system states. It is worth noting that the synchronization performance observed in the FPGA experiments is slightly degraded compared to the ideal simulation results—a discrepancy that is expected due to several practical limitations inherent in hardware implementation. First, the FPGA operates with finite-precision arithmetic (e.g., fixed-point or single-precision floating points), introducing quantization errors absent in high-precision software simulations. Second, the continuous-time PTCFZNN model must be discretized for digital realization, leading to sampling and truncation errors. Third, real-world factors such as clock jitter, signal propagation delays, power supply noise, and thermal fluctuations further degrade system accuracy. Additionally, nonlinear functions (e.g., exponential, arccot) are typically approximated using lookup tables or piecewise linear methods on FPGA to conserve resources, which may introduce deviations from their ideal mathematical forms. Importantly, no artificial noise is injected in the FPGA implementation; the observed disturbances arise naturally from these hardware non-idealities, including fixed-point quantization errors, clock jitter, and power supply fluctuations. These inherent imperfections serve as a realistic test of the PTCFZNN’s robustness under practical operating conditions. Despite these challenges, the FPGA results closely match the simulation outcomes—demonstrating rapid synchronization (within tens of milliseconds) and strong robustness—thereby validating the practical feasibility and real-time applicability of the proposed PTCFZNN model in embedded secure communication systems.

## 7. Secure Communication of the PTCFZNN-Based Chaotic System Synchronization

In practice, photoelectric signals are widely employed in environmental monitoring, industrial automation, and biomedical engineering. These signals detect variations in light intensity and convert them into measurable electrical quantities. Characterized by diverse properties and rich informational content, photoelectric signals are highly suitable for the validation and analysis of chaotic masking systems.

In this study, we simulate a photoelectric signal using the following expression:

(41) i(t)=1+0.5sin(4πt)+g

where the constant term 1 represents the baseline output level of a photoelectric sensor, and the sinusoidal component 0.5sin(4πt) (frequency: 2 Hz) models periodic variations under illumination conditions. The noise term *g* follows $N(0,\;0.1^{2})$, representing Gaussian white noise that is independent and identically distributed (i.i.d.), with a mean of zero and a standard deviation of 0.1. This simulates inevitable measurement noise and environmental interference in practical scenarios. The generated signal thus incorporates both useful information (e.g., illumination changes) and realistic noise, enhancing the validity and reliability of chaotic masking system experiments.

**Figure 5:**
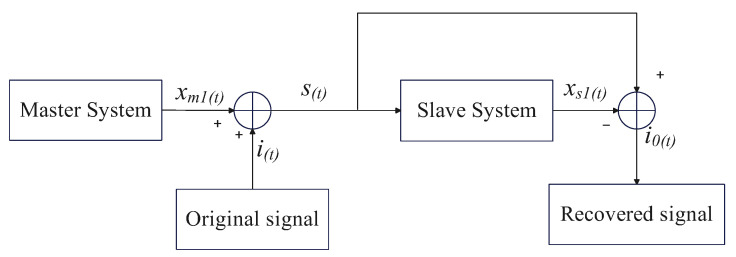
Schematic representation of a chaotic masking-based secure communication framework.

Figure 5 illustrates the core architecture of the chaotic masking-based secure communication system. The master chaotic system employs the aperiodically parameter-perturbed Chen hyper-chaotic system described earlier. The masked signal *s*(*t*) is generated by superimposing the master chaotic component $x_{m1}\left(t\right)$ onto the original photoelectric signal i(t):

(42) $$s\left(t\right)=i\left(t\right)+x_{m1}\left(t\right).$$

The recovered signal $i_{0}\left(t\right)$ is obtained using the slave chaotic component $x_{s1}\left(t\right)$, synchronized via the PTCFZNN:

(43) $$i_{0}\left(t\right)=s\left(t\right)-x_{s1}\left(t\right).$$

**Figure 6:**
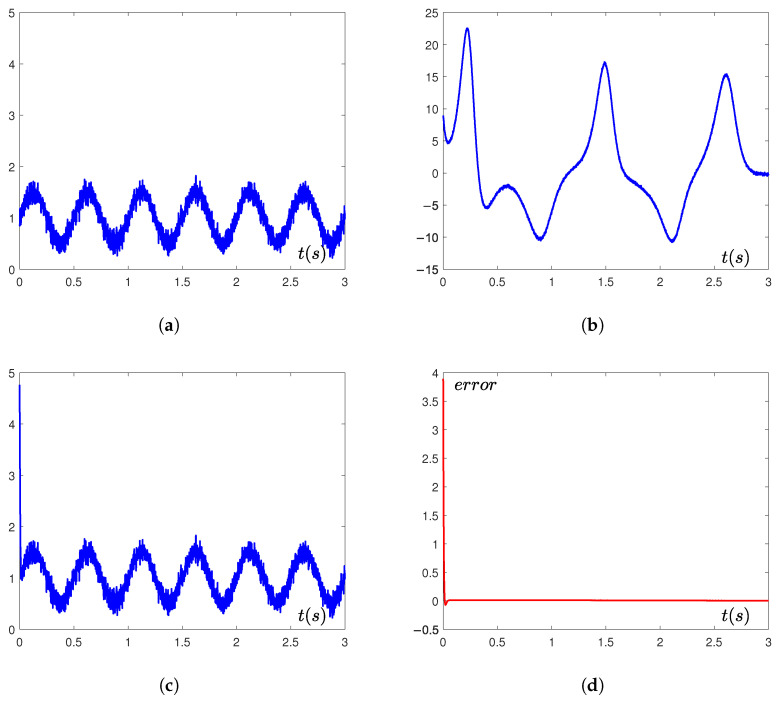
Signal encryption and recovery performance of PTCFZNN in chaotic masking communication. (**a**) Original signal. (**b**) Masked signal. (**c**) Recovered signal. (**d**) Recovery error.

The simulation results in Figure 6 demonstrate that the designed PTCFZNN-synchronized chaotic masking system achieves rapid recovery of the photoelectric signal within an exceptionally short interval, and the recovery error is almost 0.

To quantitatively characterize the masking capability, we analyzed the power ratio between the photoelectric signal (i(t)) and chaotic carrier $\left(x_{m1}\left(t\right)\right)$. The signal i(t)=1+0.5sin(4πt)+g (where (g∼$N(0,0.1^{2})))$ has three components: DC offset → $Power=1^{2}=1\;V^{2}$; sinusoidal term → $RMS=0.5/\sqrt{2}\approx 0.354\;V$ → $Power=({\left(0.354\right)}^{2}\approx 0.125\;V^{2})$; Gaussian noise → $Power=0.01\;V^{2}$. Then, total signal power → $P_{\mathrm{signal}}=1+0.125+0.01=1.135\;V^{2}$. The chaotic carrier $x_{m1}\left(t\right)$ (Figure 6) exhibits bounded dynamics with peak amplitudes ≈ ±20V under aperiodic excitation. Empirically, its mean square power is

(44) $$P_{\mathrm{chaos}}\approx \frac{1}{T}\int_{0}^{T}x_{m1}^{2}\left(t\right)dt\approx 250\;\;V^{2}$$

The critical masking ratio is thus

(45) $$\frac{P_{\mathrm{signal}}}{P_{\mathrm{chaos}}}\approx \frac{1.135}{250}=0.00454\;\left(-23.4\;\;\mathrm{dB}\right)$$

Figure 6c confirms successful recovery at this ratio. The system exhibits excellent secure communication performance, validating the efficacy of the proposed approach.

## 8. Conclusions

This paper proposes a new PTCFZNN method based on Takagi–Sugeno fuzzy control to solve the synchronization control problem of non-periodic parametrically excited chaotic systems. Theoretical analysis establishes global stability and provides an explicit, initial-condition-independent upper bound on convergence time—essential for real-time applications. Simulations demonstrate significantly enhanced convergence speed and robustness under various noise conditions, while FPGA implementation confirms hardware feasibility and real-time performance despite finite-precision effects and other non-idealities. In chaotic masking communication experiments, the system enables secure signal transmission and efficient recovery by embedding information into chaotic states, showcasing its practical utility. Compared to conventional digital communication relying on upper-layer encryption (e.g., AES, RSA), the proposed method offers inherent physical-layer security: the noise-like chaotic carriers and extreme sensitivity to initial conditions and controller parameters (e.g., $\rho_{1},\rho_{2},\sigma ,\alpha_{i}$) ensure strong key sensitivity and make eavesdropping highly difficult. These advantages—preset-time convergence, robustness, and hardware realizability—highlight PTCFZNN’s potential for secure data transmission in embedded systems, including sensor networks, bio-signal communication, and power electronics. Future work will address current limitations related to model accuracy and structural mismatches by developing adaptive parameter estimation methods, extending the framework to fractional-order systems, and conducting comprehensive security analyses (e.g., key space, information entropy, NPCR/UACI, known-plaintext attack resilience) to fully evaluate its cryptographic strength.

## Figures and Tables

**Table 1:** Convergence time analysis of four models for Chen hyper-chaotic systems in noise-free environment.

Noise Type	Noise-Free	Linear Noise	Exponential Noise	Mixed Noise
DSZNN	0.429565 s	0.481293 s	0.434673 s	0.493382 s
SEZNN	0.74097 s	1.63962 s	1.40453 s	1.68987 s
CZNN	1.6602 s	Fail	1.9917s	Fail
PTCFZNN (this work)	0.0404471 s	0.0413293 s	0.0414542 s	0.0432295 s

## Data Availability

The data generated and analyzed in this study are included in this published article or are available from the corresponding author upon request.
